# Parotidites of Malarial Origin

**Published:** 1918-05

**Authors:** 


**Parotidites of Malarial Origin.** Laurent Moreau, Le Prog. Med., Nov. 10, 1917, considers these cases fairly frequent, due to extension from the frequent stomatitis, along the duct of Steno. The condition is usually bilateral abscesses from, containing streptococci and staphylococci. Fistulae are apt to prove troublesome.

